# Intermittent hypoxia treatments cause cellular priming in human microglia

**DOI:** 10.1111/jcmm.17682

**Published:** 2023-02-23

**Authors:** Martina De Felice, Lorenzo Germelli, Rebecca Piccarducci, Eleonora Da Pozzo, Chiara Giacomelli, Anna Baccaglini‐Frank, Claudia Martini

**Affiliations:** ^1^ Department of Pharmacy University of Pisa Pisa Italy; ^2^ Department of Mathematics University of Pisa Pisa Italy

**Keywords:** CD86, HIF‐1α, HLA‐DRα, IL‐6, intermittent hypoxia, microglial priming, mild cognitive impairment, neuroinflammation, NF‐κB, obstructive sleep apnoea syndrome

## Abstract

Obstructive sleep apnoea syndrome (OSAS) is a sleep‐disordered breathing characterized by nocturnal collapses of the upper airway resulting in cycles of blood oxygen partial pressure oscillations, which lead to tissue and cell damage due to intermittent hypoxia (IH) episodes. Since OSAS‐derived IH may lead to cognitive impairment through not fully cleared mechanisms, herein we developed a new in vitro model mimicking IH conditions to shed light on its molecular effects on microglial cells, with particular attention to the inflammatory response. The in vitro model was set‐up and validated by measuring the hypoxic state, HIF‐1α levels, oxidative stress by ROS production and mitochondrial activity by MTS assay. Then, the mRNA and protein levels of certain inflammatory markers (NF‐κB and interleukin 6 (IL‐6)) after different IH treatment protocols were investigated. The IH treatments followed by a normoxic period were not able to produce a high inflammatory state in human microglial cells. Nevertheless, microglia appeared to be in a state characterized by increased expression of NF‐κB and markers related to a primed phenotype. The microglia exposed to IH cycles and stimulated with exogenous IL‐1β resulted in an exaggerated inflammatory response with increased NF‐κB and IL‐6 expression, suggesting a role for primed microglia in OSAS‐driven neuroinflammation.

## 1 INTRODUCTION

Obstructive sleep apnoea syndrome (OSAS) is a sleep‐disordered breathing, often preceding dementia and cognitive impairment.^1^ Specifically, sleep disturbance has been associated with cognitive dysfunctions, deficits in vigilance, memory and visuo‐spatial abilities,^2^ with particular regard to mild cognitive impairment (MCI) onset.^3^, ^4^ However, the exact mechanisms by which these pathologies are related to OSAS remain to be elucidated.

OSAS is characterized by recurrent episodes of intermittent hypoxia (IH),^5^, ^6^ leading to cyclic oscillations of oxygen levels (pO_2_) in arterial blood and tissues as well as establishing chronic hypoxia/reoxygenation mechanisms that effectively contribute to cellular and tissue damages.^7^ In particular, IH condition leads to endothelial dysfunction, glucose dyshomeostasis, hypertension, systemic inflammation and pathological brain alterations.^1^, ^8^

One of the main IH‐dependent mechanisms promoting cell damage is oxidative stress. Indeed, IH condition is related to an imbalance between the production and elimination of reactive oxygen species (ROS), resulting in increased ROS levels and reduced antioxidant enzymatic activity.^9^ Specifically, ROS production increases during the reoxygenation period, altering physiological cellular mechanisms.^10^

The cellular response to hypoxia is regulated by the inducible factor 1 (HIF‐1), necessary for O_2_ homeostasis maintenance.^11^ Under hypoxia,^12^ the lack of O_2_ in combination with a high ROS production reduce the degradation of HIF‐1α subunit, promoting its translocation to the nucleus where the HIF‐1α/HIF‐1β complex is formed. The binding of the HIF‐1α/HIF‐1β complex to the hypoxia response elements induces the transcription of hypoxia‐related genes.^13^, ^14^ Among these, the key regulator of inflammation nuclear factor kappa‐light‐chain‐enhancer of activated B cells (NF‐κB) has been indicated as the pivotal one^15^, ^16^, ^17^ since it sustains the interplay between hypoxia and inflammation.^18^, ^19^

Accordingly, systemic inflammation is one of the main consequences of IH, and many studies have investigated the relationship between peripheral and central nervous system (CNS) inflammation.^20^ However, whether IH directly affects brain cells, or rather it exerts indirect effects due to peripheral alterations, is not yet completely understood.

Since microglial cells are the CNS first line immune defence and contribute to CNS inflammation,^9^ the investigation of the direct IH effects on these brain cells is of high interest. Non‐human in vivo and in vitro studies have suggested that chronic IH could lead to microglial activation.^20^ To deeply investigate the inflammatory pathway modulated by IH conditions, we developed an in vitro model using human microglia cells, allowing fast pO_2_ oscillations. Reproducing the IH state in cultured cells is very challenging; over the last years, many research groups have contributed to carry out relevant models to reproduce the IH condition.^21^, ^22^, ^23^, ^24^ Herein, we cultured an immortalized human adult‐derived microglial cell line (C20) in gas‐permeable supports allowing rapid hypoxia/normoxia cycles, in order to investigate the inflammatory responses. In particular, four IH condition protocols have been considered for the current study: (i) IH involving only hypoxia/normoxia cycles; (ii) IH followed by prolonged normoxia; (iii) IH followed by prolonged normoxia and a mild inflammatory insult to mimic a concurrent pro‐inflammatory state; and (iv) IH followed by prolonged normoxia and repeated IH bout.

Overall, we provided here, for the first time in human microglia, evidence about the effects of IH in inducing high expression of typical markers of primed microglia, which in turn overreacts following a mild inflammatory exogenous stimulus. Our data reveal microglial priming as a possible mechanism in favouring chronic CNS inflammation, thus shedding light on possible mechanisms by which IH events occurring in OSAS could prompt an inflammatory state.

## 2 MATERIALS AND METHODS

### 2.1 IH in vitro model setting‐up

#### 2.1.1 Chambers design and manufacturing

**FIGURE 1:**
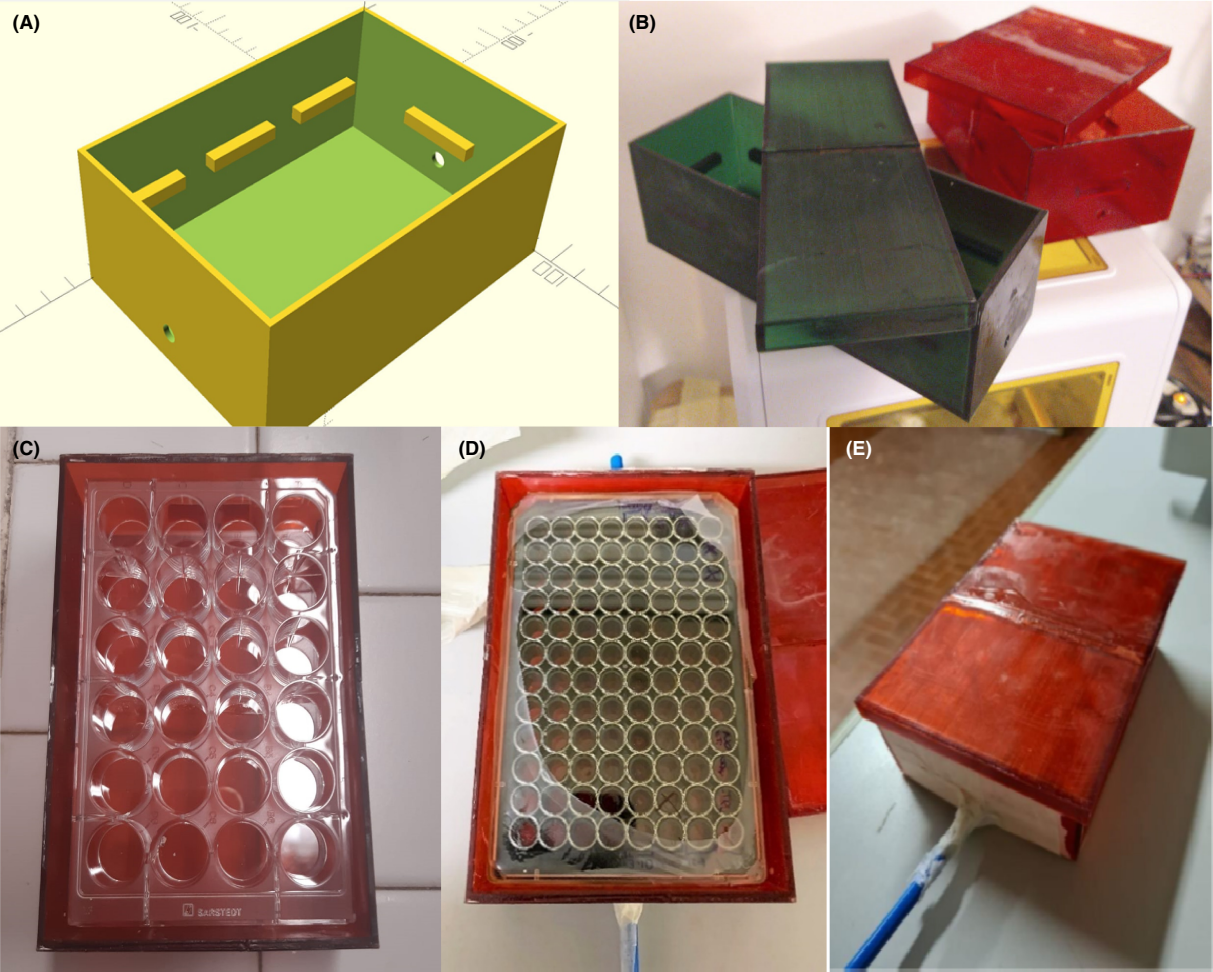
Epoxy resin chambers for IH. (A) 3D project of chamber for multiplates. (B) Petri dishes (green) or multiplates (red) chambers realized by a 3D printer. Chamber with open lid containing a 24‐well (C) or 96‐well (D) mutiplate inside. (E) Chamber for multiplate with the tightly closed lid

To perform the rapid gas exchange during IH treatments, two little chambers that were tightly closable with a lid (chamber A: 15.3 cm (l) × 5.3 cm (w) × 5 cm (h); chamber B: 13 cm (l) × 8.9 cm (w) × 5 cm (h)) were designed (Figure 1A) and realized through a 3D printer using epoxy resins (manufactured and offered by A. Vaccaro, Figure 1). The resin chambers were designed to contain three 50 mm Lumox® Petri dishes (chamber A) or one 24‐ or 96‐well Lumox® multiplate (chamber B) for cell cultures (Sarsteadt), and with specific supports to maximize gas exchange through the Lumox® gas‐permeable membranes (Figure 1B). Gasses for the hypoxia in vitro model (N_2_, O_2_ and CO_2_) were supplied by gas bottles connected to a MCQ GB 100® gas mixer (MCQ instrument©) in order to obtain the desired gas mixtures (5% CO_2_, 2% or 5% of O_2_, and 95 or 98% of N_2_). Then, the gas mixture was injected through an output gas line from the gas blender into the resin chambers for the cell culture. During treatments, the hypoxic chambers were placed in a cell culture incubator, maintaining the temperature at 37°C.

#### 2.1.2 Cell culture

C20 human microglia cells, kindly offered by Wetzel,^25^ were cultured in a DMEM F‐12 medium supplemented with 10% of Fetal Bovine Serum, 0.1 mg/ml streptomycin and 100 U/ml penicillin. Neomycin (600 μg/ml) was also added to select immortalized telomerase‐expressing cells.

#### 2.1.3 IH treatments

**FIGURE 2:**
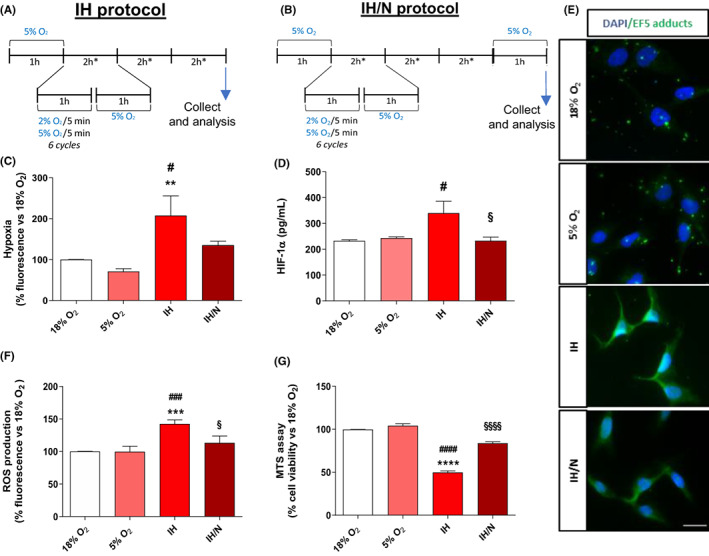
In vitro model set up and validation. (A) IH protocol and (B) IH/N protocol schemes. (C) Hypoxia detection measurements. Data are expressed as mean ± SEM of three independent experiments, statistical analysis was performed by one‐way anova followed by Bonferroni's post‐test (* vs. 5% O_2_, **p < 0.01; ^#^ vs. 18% O_2_, ^#^ *p* < 0.05). (D) HIF‐1α levels quantification. Data are expressed as mean ± SEM of two independent experiments, statistical analysis was performed by one‐way anova followed by Bonferroni's post‐test (^#^ vs. 18% O_2_, ^#^ *p* < 0.05%, ^§^ vs. IH, ^§^ *p* < 0.05). (E) Qualitative immunofluorescence analysis of EF5‐Hypoxic adducts. Scale bar = 30 μm. (F) ROS production quantification. Data are expressed as mean ± SEM of two independent experiments, statistical analysis was performed by one‐way anova followed by Bonferroni's post‐test (* vs. 5% O_2_, ***p < 0.001; ^#^ vs. 18% O_2_, ^###^ *p* < 0.001; ^§^ vs. IH, ^§^ *p* < 0.05). (G) MTS assay results. Data are expressed as mean ± SEM of two independent experiments. Statistical analysis was performed by one‐way anova followed by Bonferroni's post‐test (* vs. 5% O_2_, ****p < 0.0001; ^#^ vs. 18% O_2_, ^####^ *p* < 0.0001, ^§^ vs. IH, ^§§§§^ *p* < 0.0001).

To reproduce the recurrent episodes of IH conditions, three different protocols were performed. We considered oxygen at 5% v/v as normoxic condition, since it represents the physiological oxygen percentage in brain tissue levels,^26^ and oxygen at 2% v/v as a hypoxic state.^21^ Since almost all cell lines were usually cultured at 18% v/v of oxygen,^26^ a preconditioning phase employing oxygen treatment at 5% v/v oxygen was applied. The first protocol (IH, Figure 2A) was characterized by only hypoxia/normoxia cycles (three repeated cycles of 2 h, characterized by 1 h of IH, generated by alternation of 5 min of oxygen at 2% v/v and 5 min of oxygen at 5% v/v, for a total of 6 IH cycle/h, and 1 h of normoxic condition), mimicking the nocturnal state by which microglia underwent during OSAS. The second protocol (IH/N, Figure 2B) was performed implementing the first one with a 1 h of a prolonged normoxia condition, thus reflecting the morning awakening. The third protocol (IH/N/IL‐1β, Figure 5A) added to the IH/N condition an overnight treatment (12 h) with interleukin 1β (IL‐1β, 20 ng/ml), mimicking a concurrent exogenous inflammatory stimulus. Finally, a fourth protocol was carried out by performing an IH/N condition followed by an intermittent hypoxic stimulus, that is short IH bout (IH/N/sIH, Figure 6A).

### 2.2 Validation of the IH in vitro model

#### 2.2.1 Hypoxia detection assay

C20 cells were seeded in a 96‐well plate Lumox® (10,000 cells/well) and maintained in their complete medium. Cell hypoxic state was evaluated the day after seeding by using the EF5 Hypoxia detection kit, Alexa fluor 488® (Merck KGaA). EF5 compound in hypoxic cells forms adducts that are selectively marked by monoclonal antibody ELK3‐51 conjugated with the fluorophore Alexa fluor 488®. Briefly, cells were treated with 100 μM EF5 (2‐(2‐Nitro‐1H‐imidazol‐1‐yl)‐*N*‐(2,2,3,3,3‐pentafluoropropyl)acetamide) and then subjected to IH and IH/N protocols in serum‐free medium. In parallel, the treatment with 100 μM EF5 compound was also performed for cells maintained at 5% or 18% v/v of oxygen (controls). Then, cells were fixed with 4% Paraformaldehyde in phosphate‐buffered saline solution (pH 7.4, PBS) for 15 min and then washed three times with PBS. Cell membranes were permeabilized with 2.5% BSA and 0.1% Triton 100× in PBS, for 10 min. Then, cells were blocked in 2.5% BSA in PBS for 1 h at room temperature and then incubated with ELK3‐51 Ab (diluted 1:100) at 4°C overnight. Then, cells were washed three times with PBS. Fluorescence was quantified by using EnSightTM multimode plate reader (Ex: 485 nm, Em: 520 nm; PerkinElmer Inc) and normalized to the control at 18% v/v of oxygen.

#### 2.2.2 Immunostaining of hypoxic adducts

C20 cells were seeded in a 24‐well plate Lumox® (3500 cells/well) and maintained in their complete medium. Cells' hypoxic state was evaluated the day after seeding by using the EF5 Hypoxia detection kit, Alexa fluor 488® (Merck) as described in section 2.2.1. After washes with PBS, cells' nuclei were stained with DAPI (Merck). Then, the gas‐permeable film was cut, detached from the plate and mounted with Vectashield (Vector Labs) on a support for imaging with an epifluorescent microscope (Nikon E‐Ri). For the detection of DAPI and EF5 adducts, images from the blue and green emission channels were acquired at 40× magnification, by keeping constant exposure time and acquisition parameters for the analysis. Images background was subtracted, and a linear brightness/contrast enhancement was performed by using ImageJ (https://imagej.nih.gov/ij/). Different channels were then merged in post processing analysis of images.

#### 2.2.3 ROS quantification assay

ROS production evaluation was assessed by using the probe H_2_DCF‐DA (Molecular Probes, Invitrogen) that under ROS presence is oxidized to fluorescent 2′,7′‐dichlorofluorescein. Cells were seeded in 96‐well plate Lumox® (Sarsteadt, 10,000 cells/well) and maintained in complete culture medium. The day after seeding, cells were treated with IH, IH/N or control protocols in serum‐free medium.

After the protocols, the cells were kept in the dark for 30 min at 37°C in PBS/glucose medium containing 20 μM of H_2_DCF‐DA. Then, the solution was replaced with fresh PBS/glucose medium; fluorescence intensity was quantified by EnSightTM multimode plate reader (Ex: 485 nm; Em: 520 nm; PerkinElmer Inc.) and normalized to the control.

#### 2.2.4 HIF‐1α protein quantification

C20 cells were seeded in 50 mm Lumox® Petri dishes (200,000 cells/dish) and subjected to IH, IH/N or control protocols. After the treatments, the cells were lysed (0.2% TRIS, 0.8% NaCl, 0.07% EDTA, 0.01% Glicerol, 0.001% IGEPAL, protease inhibitor cocktail (Merck)) and sonicated in ice for 30 s. The lysates were maintained for 2 h under constant rotation at 4°C and centrifuged at 13,000*g* at 4°C. Protein DC Assay Reagent® (Biorad) was used for protein quantification according to the manufacturer's instructions. The HIF‐1α levels were assessed by high‐sensitivity ELISA kit (Cloud Clone Corp, SEA798Hu, Detection Range 0.156–10 ng/ml), by using a total amount of 20 μg of total proteins from each cell extract.

#### 2.2.5 Mitochondrial activity assay

C20 cells were seeded in a complete culture medium in 96‐well plate Lumox® (10,000 cells/well). The day after the medium was changed to starvation medium and cells were subjected to IH, IH/N or control protocols. Mitochondrial activity was evaluated by MTS assay according to the manufacturer's instructions (Promega).^27^

### 2.3 Microglial inflammatory state and priming evaluation

#### 2.3.1 Real‐time RT‐PCR

C20 cells were seeded in 24‐well plate Lumox® (80,000 cell/well) and maintained in complete culture medium. The day after, the seeding medium was changed to starvation medium and cells were treated following IH, IH/N, IH/N/IL‐1β, ΙΗ/Ν/sΙΗ or control protocols. After the treatments, cells were detached by trypsin/EDTA and total RNA was extracted by using Rneasy Mini Kit® (Qiagen). NanoDrop™ Lite Spectrophotometer (Invitrogen) was used to evaluate RNA amount and purity. Then, the RNA was retro‐transcribed to cDNA by using the iScript cDNA Synthesis Kit (Bio‐Rad). Real‐Time PCR reactions were performed using 25 ng of cDNA, 10 μl of SsoAdvanced Universal SYBR Green Supermix (Bio‐Rad) and 500 nM each of the forward and reverse primers (Table 1). Temperatures for each reaction step were as follow: 95°C for 15 s, 98°C for 30 s, and primer‐specific annealing and extension temperatures for 30 s (Table 1). Denaturation, annealing and extension phases were repeated for 40 cycles. β‐actin was used as the housekeeping gene to normalize Ct values, and 2^−ΔΔct^ method was used for mRNA expression relative quantification.

**TABLE 1:** List of primers used in Real time PCR

Gene	Primers sequence	T Annealing (°C)	Amplicon size (bp)
NF‐κB	FOR: AATGGGCTACACCGAAGCAA REV: CTGTCGCAGACACTGTCACT	56	147
IL‐6	FOR: TCCTCGACGGCATCTCA REV: TTTTCACCAGGCAAGTCTCCT	62	165
CD86	FOR:AGGCAACAATGAGCAGACCA REV: ACTATGGCTTGTTGGGTGGG	64	126
HLA‐DRα	FOR: GCTCACAAACAGCCCTGTGG REV: CCATTTCGAAGCCACGTGACA	66	102
CX3CR1	FOR: CATCACCGTCATCAGCATTGA REV: GGTAGTCACCAAGGCATTCATT	60	182
β‐Actin	F: GCACTCTTCCAGCCTTCCTTCC R: GAGCCGCCGATCCACACG	58	254

*Note*: List of sequence, amplicon size in bp, and annealing temperature of primers used in RT‐PCR. Primers were designed to amplify specifically regions between two exon and to skip intron.

#### 2.3.2 NF‐κB and interleukin release quantification

C20 cells were seeded in complete culture medium in 50 mm Lumox® Petri dishes (500,000 cells/dish) and in 24‐well plate Lumox® (80,000 cells/well) for NF‐κB and interleukin 6 (IL‐6) release quantification, respectively. The day after, the medium was changed to serum‐free medium and the cells were treated IH, IH/N or control protocols.

For NF‐κB assessment, the cells were detached and centrifugated at 300*g* for 5 min. The cellular nuclei and cytoplasm were isolated by resuspending the pellet in a subcellular fraction buffer (Sucrose 250 mM, HEPES 20 mM, KCl 10 mM, MgCl_2_ 1.5 mM, EDTA 1 mM, EGTA 1 mM, DTT 1 mM and protease inhibitor cocktail, pH = 7.4) and by centrifugation at 1000*g*, 10 min. RIPA buffer (0.5% sodium deoxycholate PBS, pH = 7.4, 1% Igepal, 0.1% SDS and protease inhibitors cocktail) was added to nuclear and cytoplasm fractions and sonicated in ice for 30 s. Lysates were maintained for 2 h under continuous rotation at 4°C and then were centrifuged at 13,000*g* at 4°C. Protein quantification was performed by using Protein DC Assay Reagent®. A total amount of 20 μg of proteins of each extract was separated by electrophoresis SDS‐PAGE, by using pre‐cast gel (4%–20%). Then, the proteins were transferred to a PVDF membrane that was incubated in blocking solution (5% non‐fat dry milk, 0.1% tween 20 in TBS pH = 8) for 2 h and overnight at 4°C with primary antibody (dilution 1:200, Santa Cruz Biotechnology, SC‐8008). The following day, the membranes were washed with TBS‐T (0.02% Tween 20 in TBS, pH = 8) and incubated for 2 h with anti‐mouse IgG HRP‐conjugated at room temperature (dilution 1:10,000, Invitrogen, 31430). The PVDF membranes were washed with TBS‐T and then the ECL substrate reagent (Bio‐Rad) was used to detect the protein bands, whose signal was visualized by ChemiDoc XRS+ System (Bio‐Rad). Stain‐Free technology was employed as a normalization tool in Western blot analysis, avoiding the use of housekeeping proteins.^28^

For IL‐6 assessment after IH protocols, the conditioned medium was collected from each well, centrifuged for 5 min at 20,000*g* at 4°C and 100 μl of supernatant was used for IL‐6 quantification by using a commercial ELISA kit (Cloude Clone Corp; SEA079Hu, detection range: 7.8–500 pg/ml).

### 2.4 Statistical analysis

All results are presented as means ± SEMs from at least three independent experiments. Statistical analysis was performed as indicated in each graph caption by using Graphpad Prism Software (GraphPad Software Inc). For two column analysis, an unpaired *t*‐test was employed, while for more than two column analysis, a one‐way anova was used. A *p* value lower than 0.05 (*p* < 0.05) was considered statistically significant.

## 3 RESULTS

### 3.1 In vitro model set‐up and microglial response characterization

To verify whether the set‐up model was effectively able to establish and maintain a hypoxic environment, C20 cells were exposed to IH treatments (Figure 2A,B) and analysed for a hypoxic state by measuring both the formation of hypoxic‐related adducts and the levels of HIF‐1α protein. For both, no significant differences were observed between cells cultured with 18% compared to 5% of O_2_ (Figure 2C–E). Conversely, fluorescence intensity and HIF‐1α levels dramatically increased after IH treatments confirming that those protocols causes cell hypoxic state. Notably, the hypoxic condition was reduced with the IH/N protocol. To further explore and validate the in vitro model, ROS production was measured in C20 cells by means of H_2_DCF‐DA fluorescence probe. As expected, the data obtained showed no significant differences between ROS production in cells maintained at 18% and 5% of oxygen (Figure 2F). On the contrary, ROS levels significantly increased after the IH protocol (Figure 2F), while the IH/N protocol led to their reduction, suggesting the partial restoration of normoxia.

Moreover, to characterize C20 cells' response to IH or IH/N treatments, the MTS assay, which is useful to evaluate mitochondrial metabolic activity,^29^ was performed. Results suggested a robust reduction of cellular metabolic activity following the IH protocol compared to cells maintained at 5% oxygen, whereas metabolic activity reduction seemed to be less evident after IH/N (Figure 2G). Trypan blue exclusion assay was also performed to further verify cells' viability; it showed that cells' viability was not affected by any of the treatments performed (data not shown), and thus, MTS assay results specifically indicate a reduction of cells metabolic activity.

Notably, all data suggested that culturing human microglial cells at O_2_ 5% did not affect any of the evaluated parameters and, since in vivo studies reported this condition as the physiological human pO_2_ at brain level,^26^ we assumed 5% of O_2_ as normoxia (N) for human microglial cells.

### 3.2 Microglial inflammatory state evaluation

It is still not clear whether the microglial activation in OSAS is an indirect mechanism mainly due to peripheral inflammatory factors reaching the CNS, or whether the IH condition could directly affect microglial activation. For this reason, the effects of IH and IH/N on inflammatory processes were assessed in our microglial model to explore the degree of microglial activation that this condition could lead to.

**FIGURE 3:**
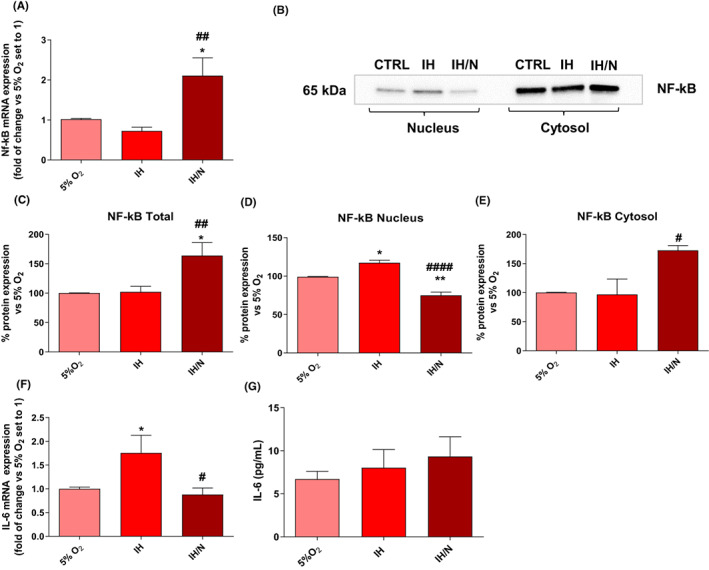
Analysis of inflammatory markers after IH or IH/N protocols. (A) NF‐κB mRNA expression. Data are expressed as mean ± SEM of three independent experiments. Statistical analysis was performed by one‐way anova followed by Bonferroni's post‐test (* vs. 5% O_2_, *p < 0.05, ^#^ vs. IH, ^##^ *p* < 0.01). (B) NF‐κB protein analysis by Western blot in nuclear and cytosolic fractions. (C) Total amount of NF‐κB levels in cell lysates. (D, E) Amount of NF‐κB levels in nuclear and cytosolic fractions. Data are expressed as mean ± SEM of two independent experiments. Statistical analysis was performed by one‐way anova followed by Bonferroni's post‐test (* vs. 5% O_2_ *p < 0.05, **p < 0.01, ^#^ vs. IH, ^#^ *p* < 0.05, ^####^ *p* < 0.0001). (F) IL‐6 mRNA expression by RT‐PCR. Data are expressed as mean ± SEM of three independent experiments. Statistical analysis was performed by one‐way anova followed by Bonferroni's post‐test (* vs. 5% O_2_, *p < 0.05, ^#^ vs. IH, ^#^ *p* < 0.05) (G) IL‐6 release quantification by ELISA Kit. Data are expressed as mean ± SEM of two independent experiments. Statistical analysis was performed by one‐way anova followed by Bonferroni's post‐test.

Firstly, given the positive modulation of NF‐κB by the hypoxic condition and oxidative stress, the NF‐κB mRNA and nucleus/cytosol protein distribution were evaluated to understand whether IH or IH/N could determine an increase in protein expression and activation. As shown in Figure 3A,C, NF‐κB mRNA and total protein expression increased only following the IH/N protocol. However, the analysis of nuclear and cytosolic fractions showed a significant increase in the nuclear translocation of NF‐κB only after the IH treatment (Figure 3D,E). Notably, IH/N dramatically increased the NF‐κB cytosolic fraction and concomitantly decreased the nuclear translocation suggesting that the IH‐mediated inflammatory conditions could be evaded by maintaining IH cells in prolonged normoxia. As NF‐κB is a known transcription factor of pro‐inflammatory genes, such as the IL‐6, mRNA expression and protein release were evaluated after treatments with IH protocols. Data showed that, although IH increased the levels of IL‐6 mRNA (Figure 3F) compared to control or IH/N, the IL‐6 protein release did not significantly occur (Figure 3G).

Overall, these data suggest that IH is able to promote an initial activation of microglia that is ultimately ineffective in promoting a real inflammation, as suggested by the unchanged levels of IL‐6 released. Otherwise, a short recovery period of normoxia (IH/N) was able to completely counteract the accumulation of NF‐κB in the nucleus and the increase of IL‐6 mRNA levels, confirming how microglia are highly sensitive to oxygen fluctuations induced by our model.

### 3.3 Intermittent hypoxia increased the expression of markers related to primed microglia

**FIGURE 4:**
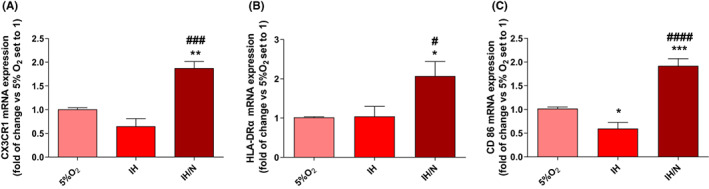
Analysis of microglial membrane marker. CX3CR1 (A), HLA‐DRA (B), CD86 (C) mRNA levels obtained by RT‐PCR. Data are expressed as mean ± SEM of three independent experiments. Statistical analysis was performed by one‐way anova followed by Bonferroni's post‐test (* vs. 5% O_2_, *p < 0.05, **p < 0.01, ***p < 0.001; ^#^ vs. IH, ^#^ *p* < 0.05, ^####^ *p* < 0.0001).

Although C20 cells did not appear to be activated after the IH and IH/N protocols, IH conditions could give rise to other kind of response mechanisms due to the overexpression of basal NF‐κB levels, such as the sensitization state in microglia, called *priming*. Microglial priming is a phenomenon that occurs in microglial cells after a first inflammatory signal and a not completely recover^30^, ^31^; the overexpression of basal NF‐κB levels makes the microglia more susceptible to a secondary inflammatory stimulus that triggers an exaggerated inflammatory response.^30^ Since studies in the literature have reported an NF‐κB dependent upregulation of priming genes,^32^ our data prompted us to investigate the mRNA expression of specific receptor markers typical of primed microglia.^30^ Thus, the mRNA expression of HLA‐DRα, CD86 and CX3CR1^33^, ^34^, ^35^ was evaluated in C20 cells treated with IH, IH/N or control protocols. As shown in Figure 4, after IH, no changes were detected for HLA‐DRα and CX3CR1, indicating that the phenotype had not changed, yet. On the contrary, mRNA levels of all the analysed markers significantly increased after IH/N treatment (Figure 4A–C) compared to IH condition as well as to normoxia (5% O_2_), effectively suggesting the capacity of IH to induce the human microglia priming.

### 3.4 Microglial priming evaluation

**FIGURE 5:**
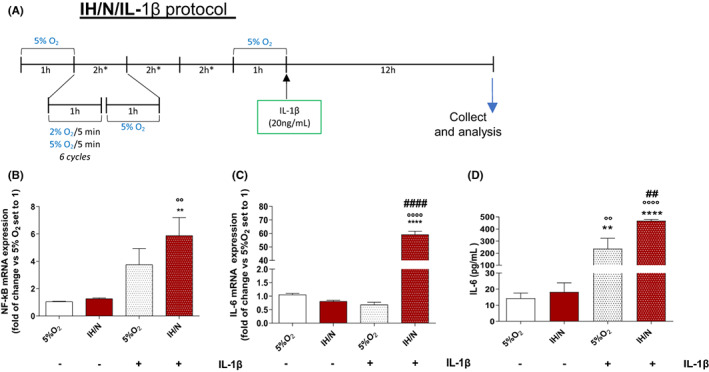
Microglial Priming evaluation after IH/N treatment and IL‐1β administration. (A) IH/N protocol with IL1‐β exogenous treatment scheme. (B) NF‐κB and IL‐6 (C) mRNA expression analysis by RT‐PCR. Data are expressed as mean ± SEM of two independent experiments. Statistical analysis was performed by one‐way anova followed by Bonferroni's post‐test (* vs. 5% O_2_ (−) IL1‐β, **p < 0.01, ****p < 0.0001; ^#^ vs. 5% O_2_ (+) IL1‐β, ^####^ *p* < 0.0001; ° vs. IH/N (−) IL1‐β, °°*p* < 0.01, °°°°*p* < 0.0001. (D) IL‐6 protein levels assessed by ELISA kit. Data are expressed as mean ± SEM of two independent experiments. Statistical analysis was performed by one‐way anova followed by Bonferroni's post‐test (* vs. 5% O_2_ (−) IL1‐β, **p < 0.01, ****p < 0.0001; ^#^ vs. 5% O_2_ (+) IL1‐β, ^##^ *p* < 0.01; ° vs. IH/N (−) IL1‐β, °°*p* < 0.01, °°°°*p* < 0.0001).

**FIGURE 6:**
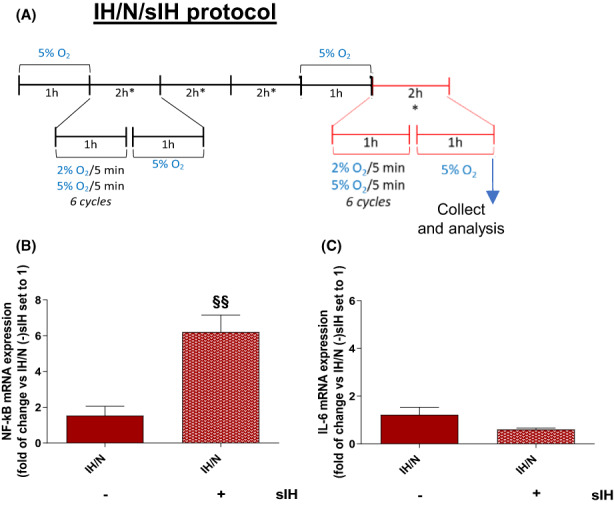
Analysis of inflammatory markers after IH/N treatment and subsequent sIH bout. (A) IH/N protocol with short IH (sIH) treatment scheme. (B) NF‐κB and IL‐6 (C) mRNA expression analysis by RT‐PCR. Data are expressed as mean ± SEM of two independent experiments. Statistical analysis was performed by Unpaired Student *t*‐test (^§^ vs. IH/N (−) sIH, ^§§^ *p* < 0.01).

The results obtained, suggesting that IH followed by normoxia might lead to a primed state of the microglia, prompted us to evaluate the microglial priming process during the normoxia recovery following IH.^30^, ^36^ Therefore, to effectively prove the microglial primed state, IH/N C20 cells were treated for 12 h with 20 ng/ml of IL‐1β (IH/N/IL‐1β protocol, Figure 5A). Levels of NF‐κB and IL‐6 were assessed and compared to cells maintained in the normoxic condition, treated (+) or not (−) with IL‐1β.^37^ Moreover, in order to investigate whether IH‐mediated priming could influence the microglia responses to subsequent IH exposures, an additional IH bout (IH/N/sIH; Figure 6A) was carried out.

Firstly, the results on mRNA levels (Figure 5B,C) showed that prolonged normoxia (IH/N without IL‐1β) did not affect the microglial activation, with no difference in NF‐κB and IL‐6 mRNA levels compared to 5% O_2_. Accordingly, IL‐6 protein levels did not statistically differ from normoxia (Figure 5D). These results further confirm how the IH‐driven microglial activation is actually a reversible state.

In IH/N cells, the IL‐1β treatment increased the NF‐κB mRNA levels of about sixfold in comparison with untreated IH/N cells (Figure 5B). A huge increase was also found for IL‐6 mRNA in IH/N/IL‐1β cells (Figure 5C), accordingly with the level of IL‐6 protein (Figure 5D). Notably, following IL‐1β treatment, normoxic cells showed higher levels of IL‐6 protein than untreated normoxic ones, although not differences were revealed in the related mRNA expression, probably due to the timing governing the correlation between mRNA production and protein expression. The obtained data corroborated the hypothesis that IH effectively induces a state of priming, which, in the presence of certain inflammatory stimulus, even mild one, can cause an inflammatory hyper‐response that could determine the insurgence of chronic neuroinflammatory processes.

Furthermore, an additional IH bout was carried out to investigate its ability to affect primed microglia inflammatory response (IH/N/sIH; Figure 6A). Our data showed that IH/N/sIH treatment increased NF‐κB mRNA levels compared to sIH untreated cells (Figure 6B), while no significant changes were found in IL‐6 mRNA expression (Figure 6C), suggesting that an additional IH bout could not be considered a pro‐inflammatory inducer but, as we demonstrated in IH/N/IL1β cells, specific exogenous inflammatory stimuli are necessary to activate an inflammatory response in primed microglia.

## 4 DISCUSSION

In recent years, OSAS has been considered to be involved in cognitive impairment insurgence and to be responsible of a variety of cognitive deficits.^38^ However, how OSAS may led to cognitive dysfunctions remains unclear. Continue Positive Air Pressure treatment represents the OSAS gold standard therapy, and it has been demonstrated to improve the cognitive dysfunctions in OSAS patients.^39^, ^40^ For this reason, oxygen level oscillations provoked by IH conditions occurring in brain tissue could represent a possible link between OSAS and cognitive impairment.^41^ Indeed, the IH state has been demonstrated to induce complex effects in CNS, by impacting microglia in different ways, especially by triggering inflammation.^20^ Uncontrolled microglial activities may cause neuronal cell injury that could lead to cognitive impairment associated with IH, although the related pathological mechanisms have not be fully elucidated, yet.

In this scenario, the development of an adequate in vitro model is essential to study MCI pathogenesis molecular mechanisms related to IH condition. Accordingly, we have developed an in vitro model to study the effects of IH on human microglia C20 cell line, since microglial cells play a key role in CNS inflammation and represent the first line of immune defence for the CNS environment.^9^

The in vitro model has been conceived to mimic IH on cell cultures with minimal gas consumption and optimal gas exchanges, by using gas‐permeable supports. The cell treatment protocols were chosen to reproduce the nocturnal IH condition occurring in OSAS patients (IH) and the awakening state that immediately follows the IH nocturnal condition (IH/N). We set up IH cycles considering switching from a normoxic (5% O_2_) to a hypoxic (2% O_2_) condition, thus reproducing the oscillations of desaturation levels typical of severe sleep apnoea syndrome.^21^, ^27^ Herein, for the first time, we analysed human microglial cell responses at 5% O_2_, considered as the normoxic condition. Thus, to verify whether the 5% O_2_ could reproduce the normal oxygen state of microglia cell culture, this condition was compared to the 18% O_2_ by analysing hypoxia susceptible markers. As expected, no differences were detected for the hypoxia state and HIF‐1α levels between 5% and 18% of O_2_, confirming that 5% O_2_ condition can be considered as normoxia for human microglial cells.

Furthermore, the hypoxia susceptible markers were also assessed after IH and IH/N protocols, obtaining increased hypoxic adducts formation and HIF‐1α concentration. It is well known that in a hypoxic condition HIF‐1α accumulates in cytosol, then it translocates into the nucleus, thus inducing the transcription of HIF‐target genes. Increased HIF‐1α levels counteract the negative effects on mitochondrial metabolism caused by lower oxygen levels.^42^ In this context, our results confirmed that the IH treatment led to a deficiency of oxygen, increased ROS production, and reduced mitochondrial activity in microglial cells that is partially reverted after a normoxic recovery (IH/N).

Then, the model was employed to evaluate the IH‐related microglia responses. Since the majority of literature focused on the IH‐dependent ROS generation responsible for microglial activation,^9^, ^43^ we investigated here inflammatory markers. Previous data have shown NF‐κB activation in BV‐2 cells^44^ following IH treatments. Moreover, in non‐microglial cells, it has been demonstrated that the NF‐κB pathway is strongly triggered by IH conditions. Nevertheless, to the best of our knowledge, no data have been reported in studying the direct effects of IH on NF‐κB activation in human microglia. Therefore, we focused on the evaluation of NF‐κB pathway activation.

Our data showed an increased NF‐κB protein translocation into the nucleus after the IH treatment. However, following a normoxic restoration period, the NF‐κB protein translocation is critically reduced, while the protein levels in the cytosol raised; also, the NF‐κB mRNA expression resulted to be increased. Thus, it seems that the IH treatment induced the NF‐κB pathway activation; nevertheless, IH state was not able to exert a proper microglial inflammatory response, as increased IL‐6 mRNA levels have been observed after IH treatment, although a statistically significant cytokine release from cells has not been achieved.

Although microglia did not appear to be fully activated after IH, this treatment can almost lead to the NF‐κB translocation; in this regard, some researchers have reported the NF‐κB dependent upregulation of those genes responsible for the process of cellular *priming* in microglia of aging mice.^32^

The existence of a peculiar microglial phenotype that occurs after a primary stimulus insult has been described in a murine model,^45^, ^46^ in which the cells maintain an activated status instead of returning to the resting state. The term ‘microglial priming’ has been used to refer to the phenotype assumed by microglial cells after a primary insult leading the primed cells to generate an exaggerated inflammatory response after a second inflammatory stimulus.^30^, ^47^, ^48^

For this reason, we have performed an investigation of HLA‐DRα, CD86 and CX3CR1 microglial priming markers transcriptional levels, highlighting an increase in their expression after the IH/N treatment.^33^, ^47^, ^49^ Similar results about increased expression of MHCII and CD86 were obtained following in vivo chronic treatment with IFN‐γ, a known stimulus triggering microglial priming process.^50^

Therefore, to verify the priming hypothesis, we tried to mimic a mild inflammatory stimulus after the IH/N protocol by treating cells with IL‐1β; increased NF‐κB, IL‐6 mRNA and IL‐6 cytokine release levels have demonstrated to be compatible with the condition of exaggerated microglial response that characterize microglial priming. Notably, our results are in line with literature data reporting a priming process in ex‐vivo microglia from stress‐induced rats, resulting in an exaggerated inflammatory response after lipopolysaccharide treatment.^51^ Furthermore, the evaluation of the inflammatory pathway activation after a short bout of IH (sIH), following IH/N protocol, confirms that repeated IH challenging is not sufficient per se to induce neuroinflammation and that exaggerated response of primed microglia occurs only after an exogenous inflammatory stimulus.

Overall, the obtained data show for the first time that the IH condition is not enough to induce the human microglia pro‐inflammatory state, even though during the following normoxia period primed microglia could overreact under a mild inflammatory stimulus. Such priming could be a possible cause of the impaired brain functions arising in neurodegenerative diseases, and, in this light, it could be pivotal to understanding the link between OSAS and cognitive impairment insurgence.

## AUTHOR CONTRIBUTIONS

**Martina De Felice:** Conceptualization (equal); data curation (equal); formal analysis (equal); investigation (lead); methodology (equal); software (equal); validation (equal); visualization (equal); writing – original draft (equal); writing – review and editing (equal). **Lorenzo Germelli:** Conceptualization (equal); data curation (equal); formal analysis (equal); investigation (equal); methodology (lead); software (equal); validation (equal); visualization (equal); writing – original draft (equal); writing – review and editing (equal). **Rebecca Piccarducci:** Conceptualization (equal); data curation (equal); formal analysis (lead); investigation (equal); methodology (equal); software (equal); validation (equal); visualization (equal); writing – original draft (equal); writing – review and editing (equal). **Eleonora Da Pozzo:** Conceptualization (lead); project administration (lead); supervision (lead); writing – original draft (equal); writing – review and editing (equal). **Chiara Giacomelli:** Resources (equal); writing – review and editing (equal). **Anna Baccaglini‐Frank:** Funding acquisition (lead); writing – review and editing (equal). **Claudia Martini:** Funding acquisition (lead); project administration (equal); writing – review and editing (equal).

## CONFLICT OF INTEREST STATEMENT

The authors declare no conflict of interest.

## Data Availability

The data that support the findings of this study are available from the corresponding author upon reasonable request.
